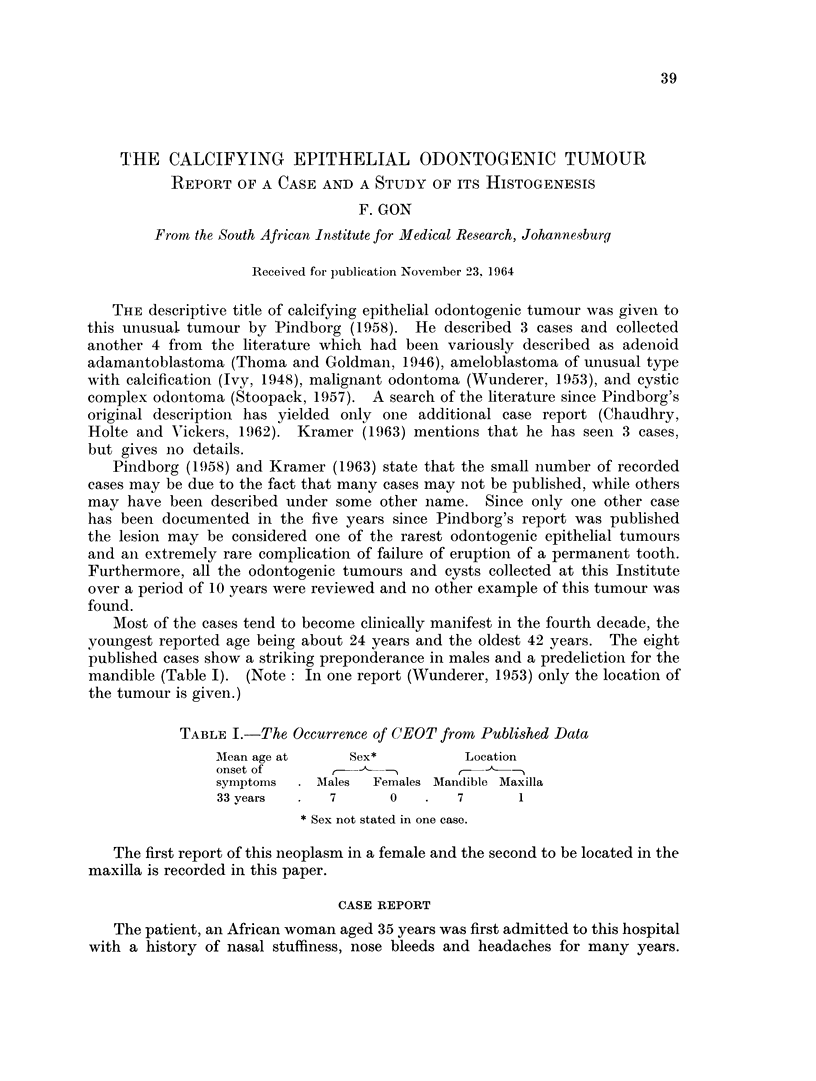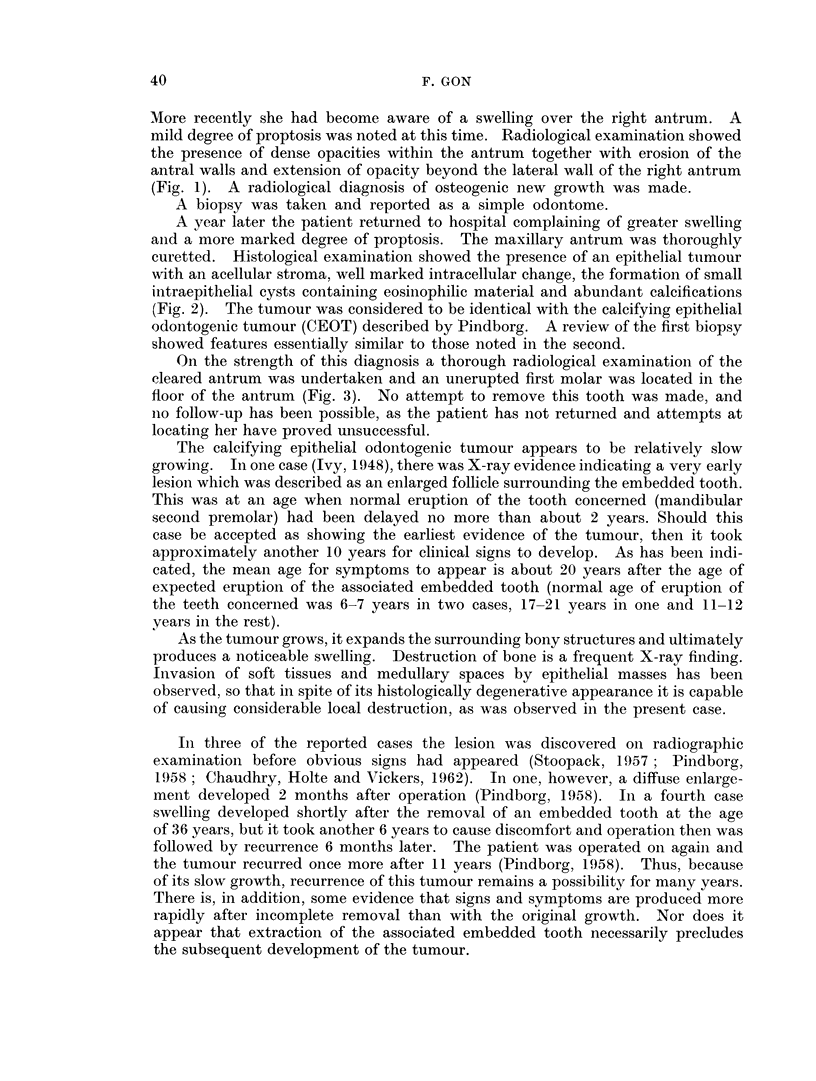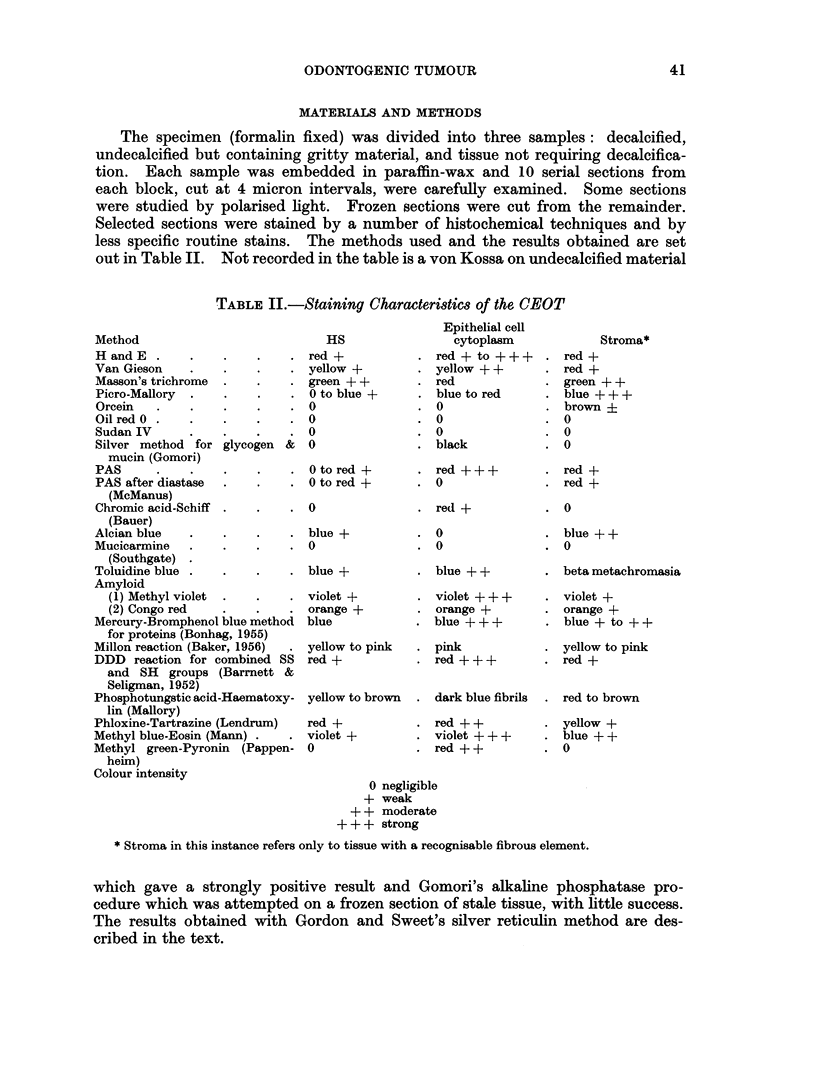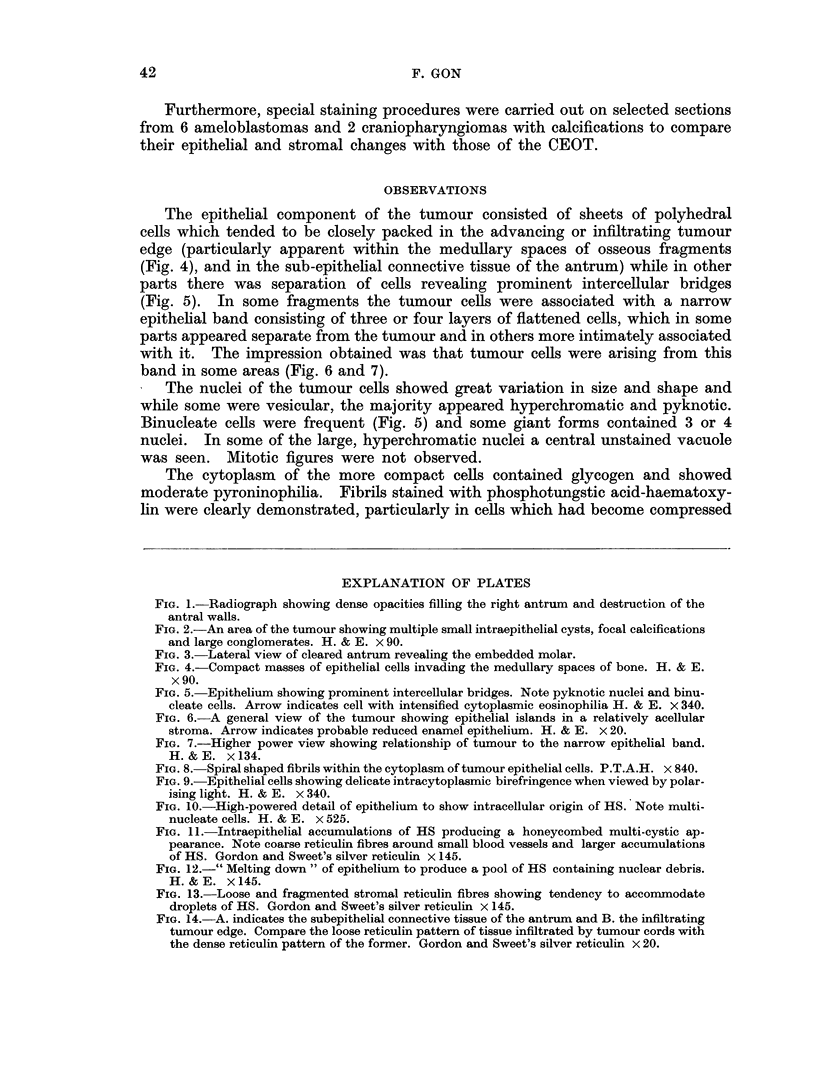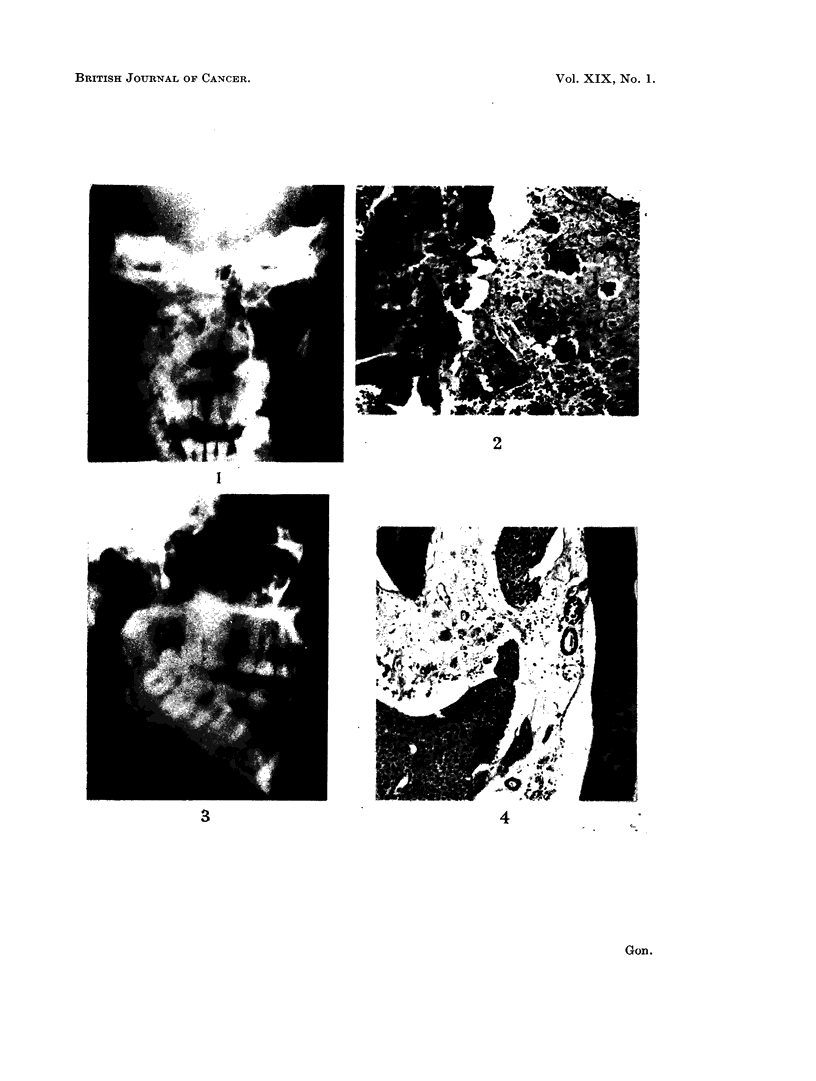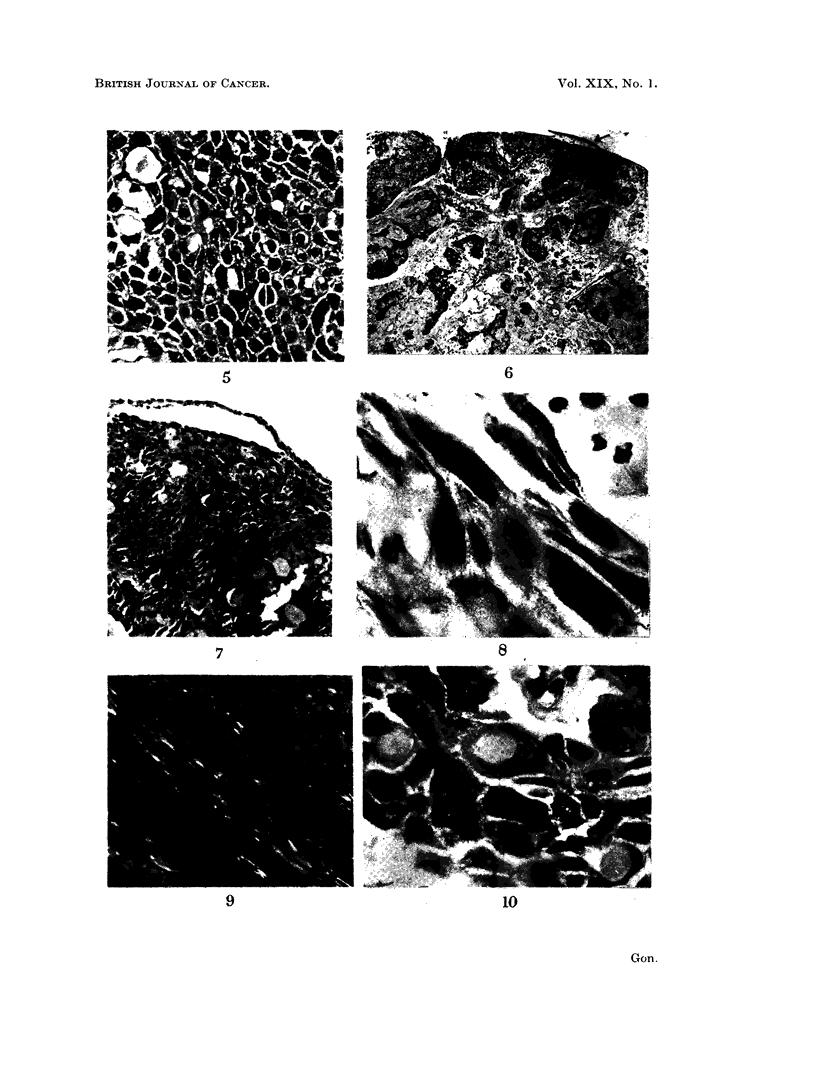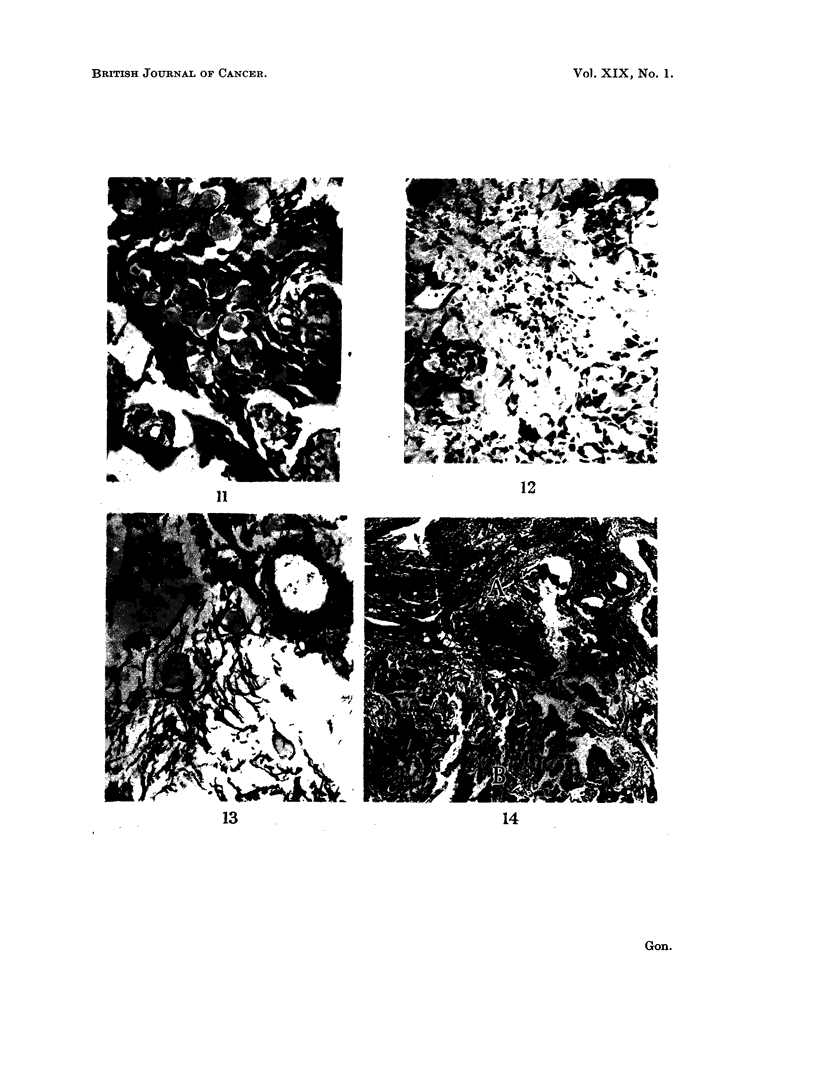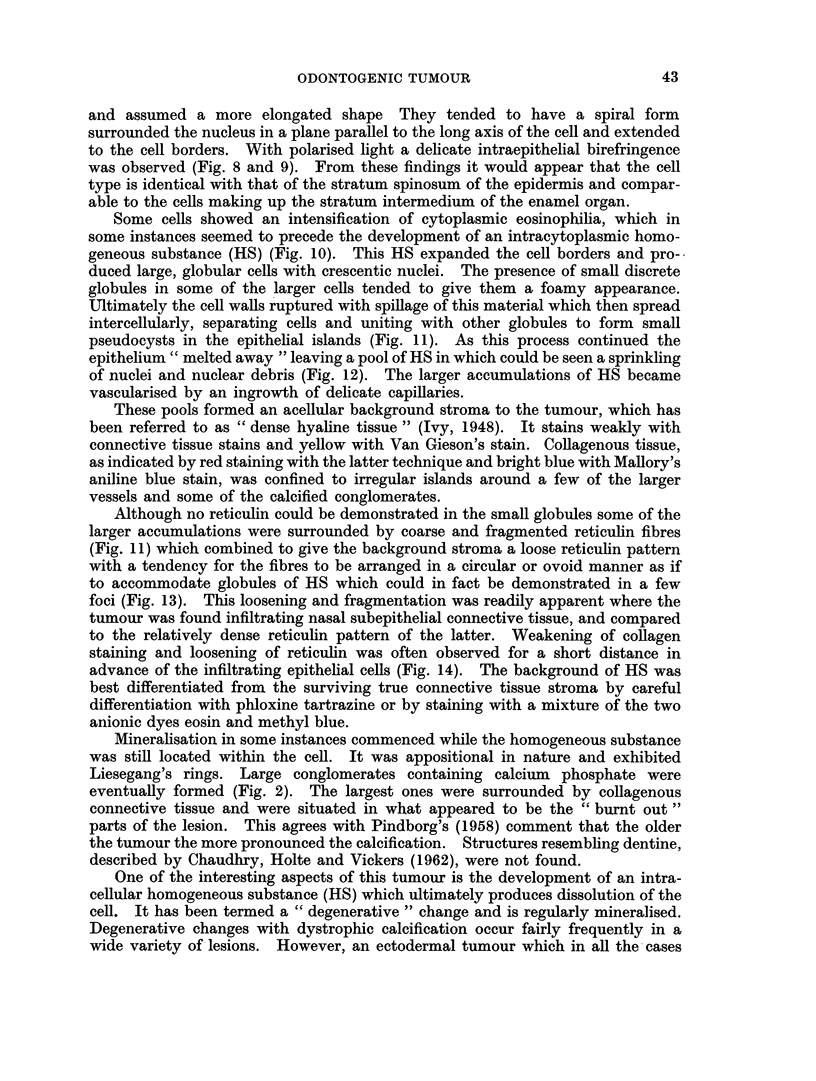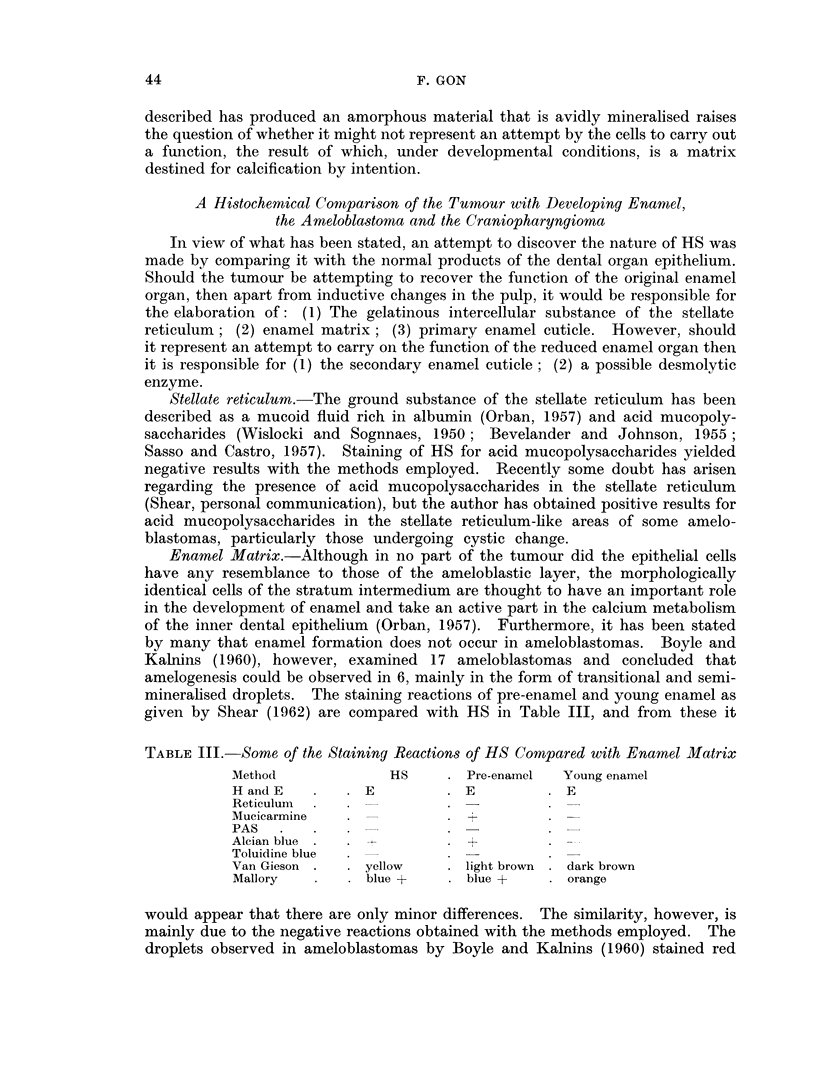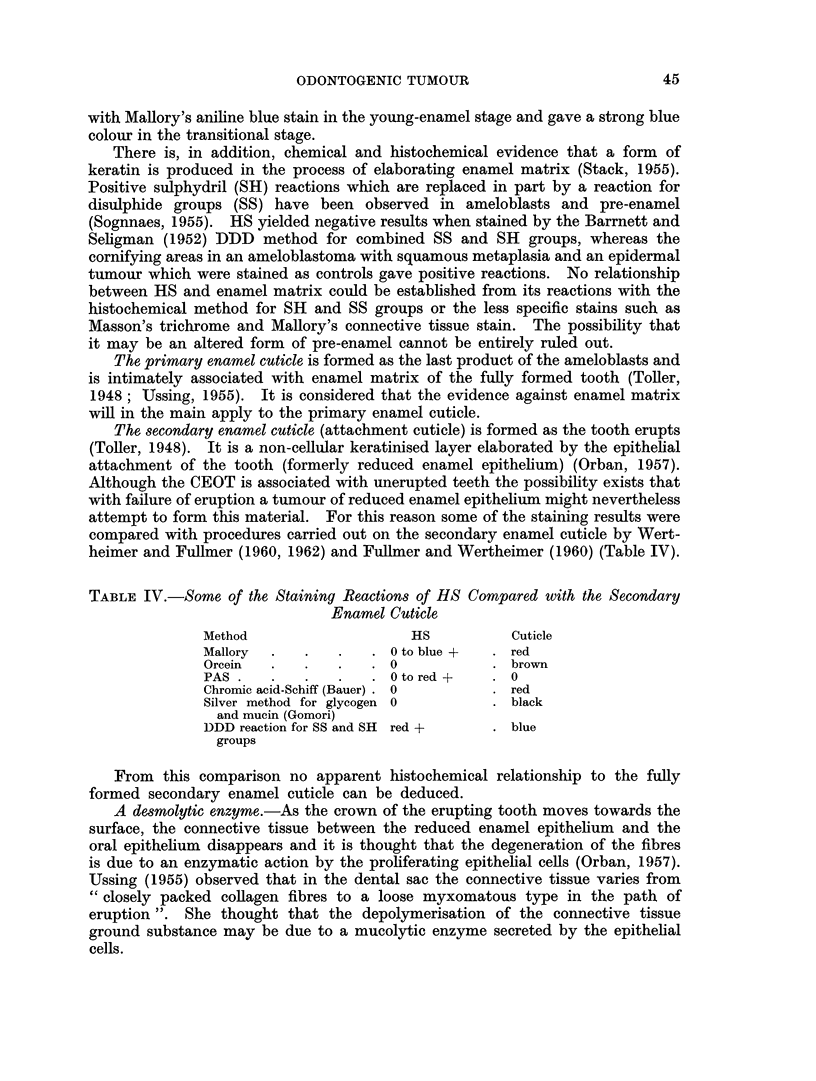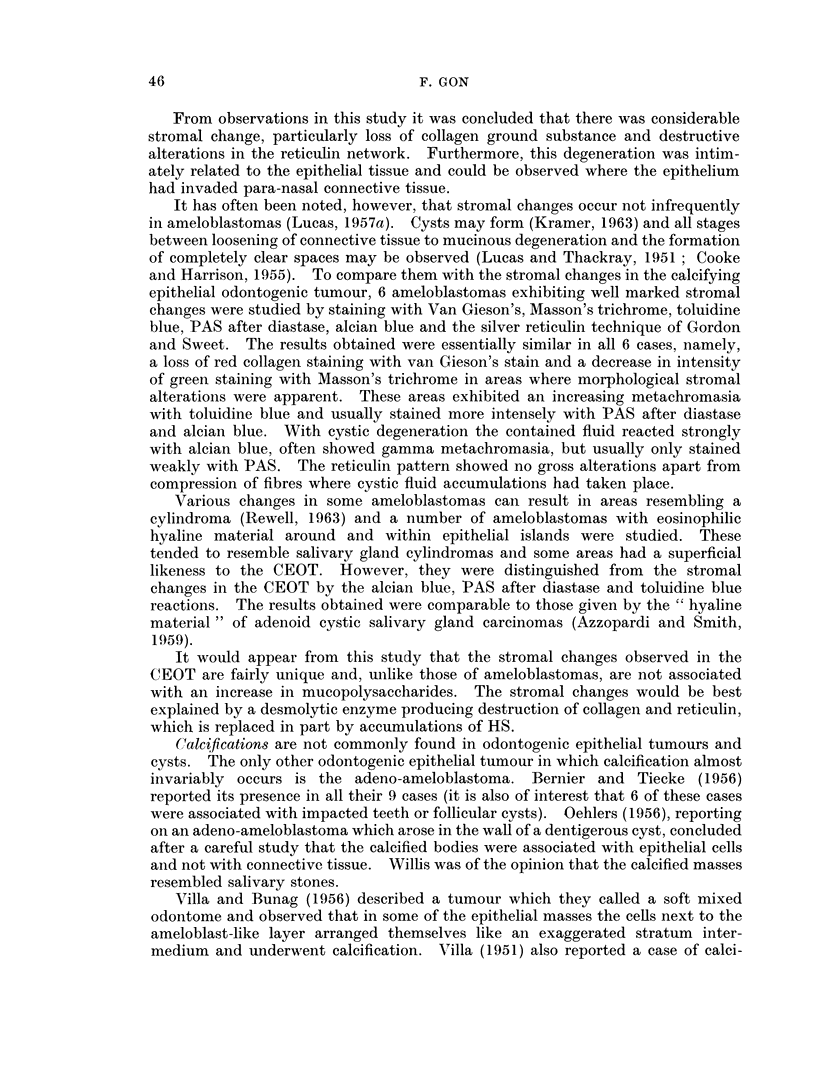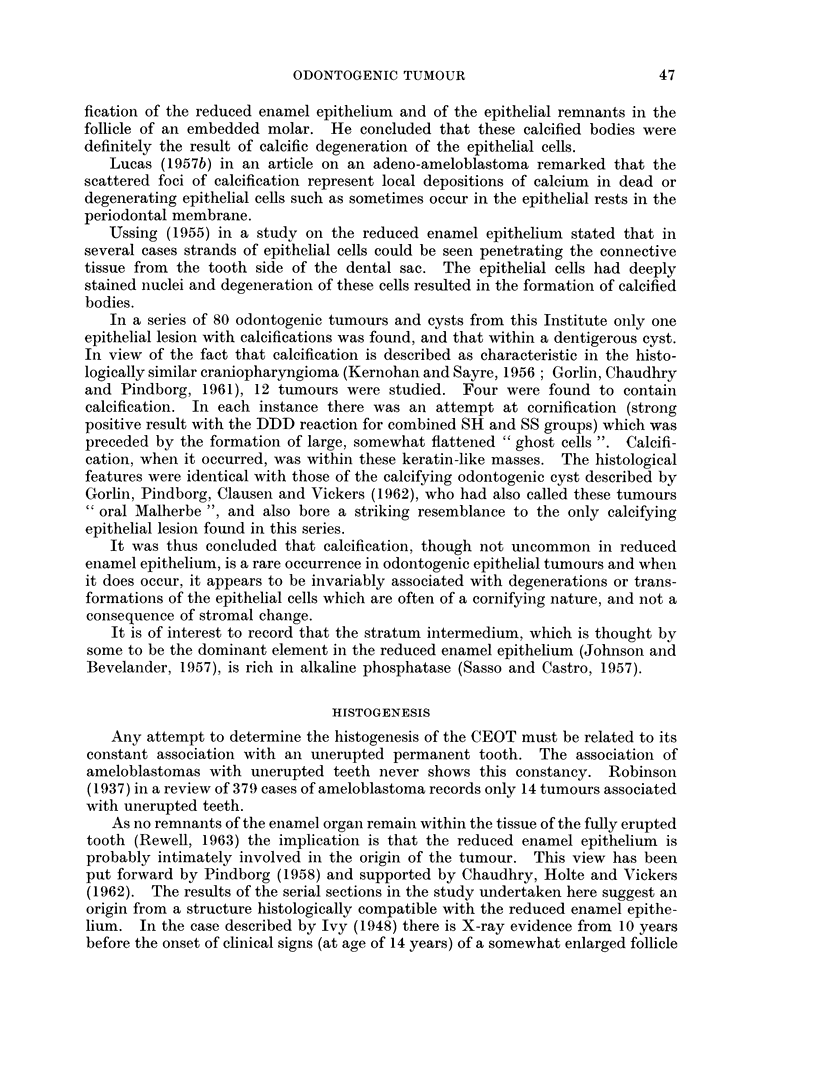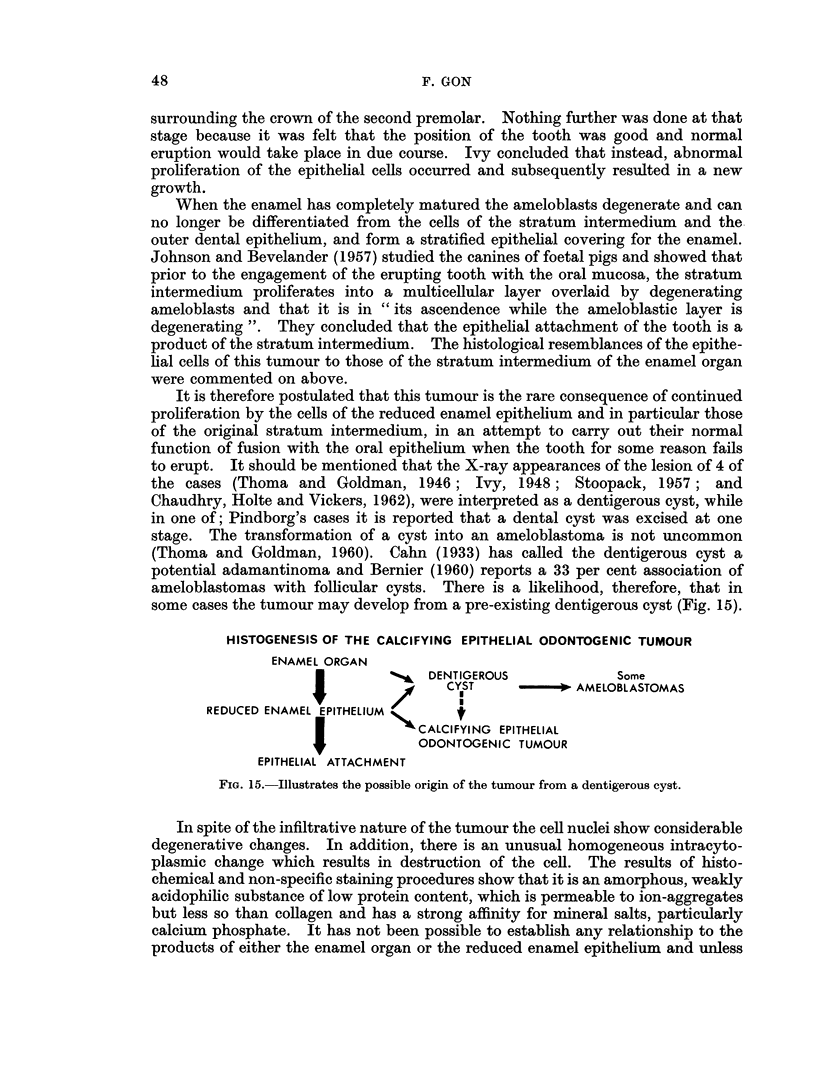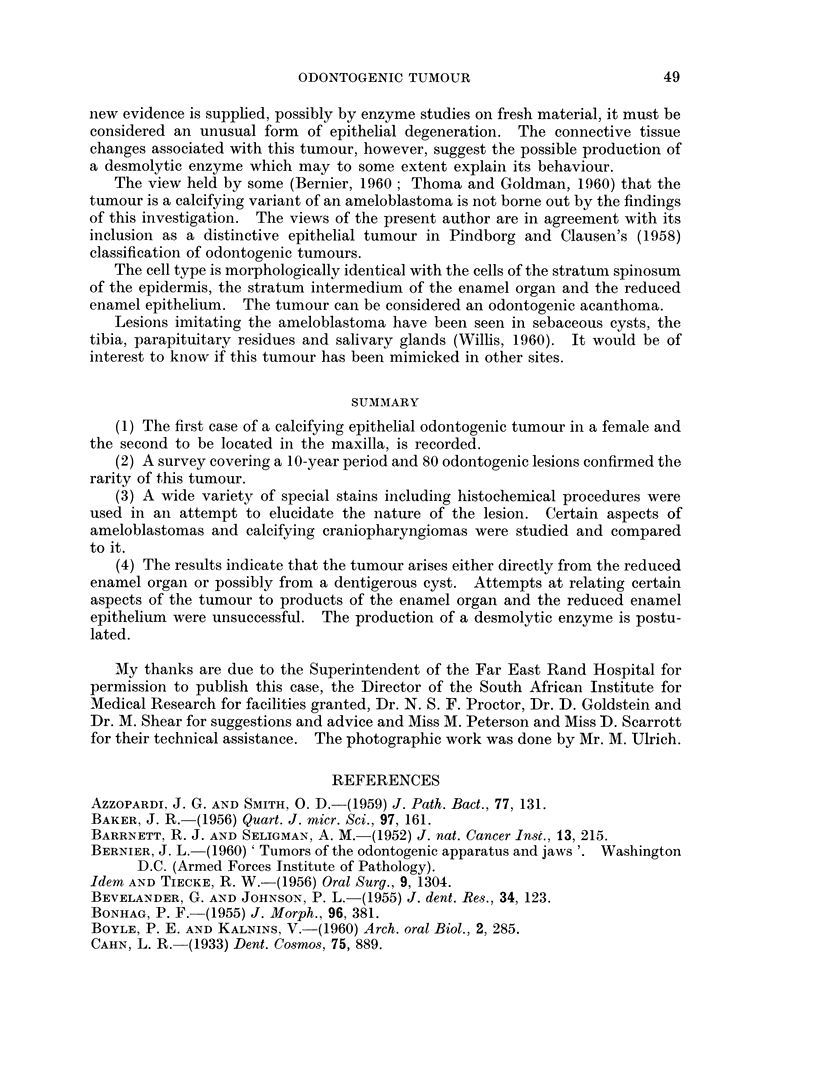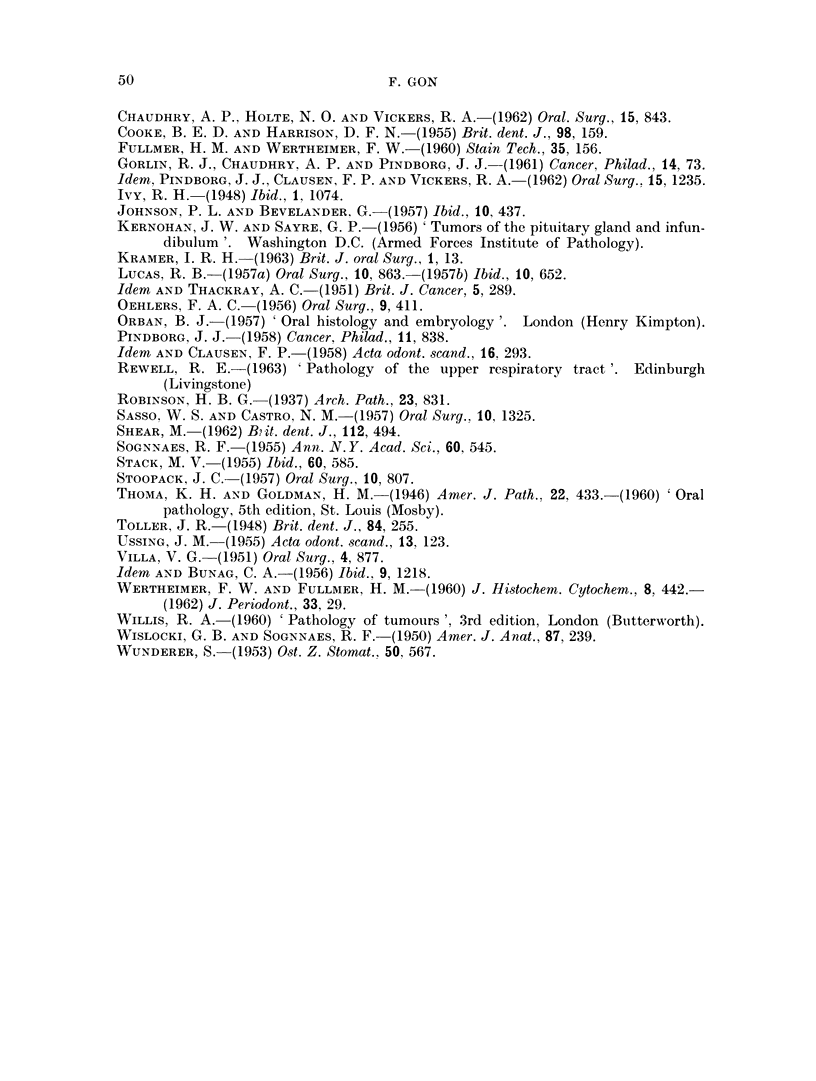# The Calcifying Epithelial Odontogenic Tumour

**DOI:** 10.1038/bjc.1965.5

**Published:** 1965-03

**Authors:** F. Gon

## Abstract

**Images:**


### THE CALCIFYING EPITHELIAL ODONTOGENIC TUMOUR

### Report of a Case and a Study of its Histogenesis

F. GON

*From the South African Institute for Medical Research, Johannesburg*

Received for publication November 23, 1964

The descriptive title of calcifying epithelial odontogenic tumour was given to this unusual tumour by Pindborg (1958). He described 3 cases and collected another 4 from the literature which had been variously described as adenoid adamantoblastoma (Thoma and Goldman, 1946), ameloblastoma of unusual type with calcification (Ivy, 1948), malignant odontoma (Wunderer, 1953), and cystic complex odontoma (Stoopack, 1957). A search of the literature since Pindborg's original description has yielded only one additional case report (Chaudhry, Holte and Vickers, 1962). Kramer (1963) mentions that he has seen 3 cases, but gives no details.

Pindborg (1958) and Kramer (1963) state that the small number of recorded cases may be due to the fact that many cases may not be published, while others may have been described under some other name. Since only one other case has been documented in the five years since Pindborg's report was published the lesion may be considered one of the rarest odontogenic epithelial tumours and an extremely rare complication of failure of eruption of a permanent tooth. Furthermore, all the odontogenic tumours and cysts collected at this Institute over a period of 10 years were reviewed and no other example of this tumour was found.

Most of the cases tend to become clinically manifest in the fourth decade, the youngest reported age being about 24 years and the oldest 42 years. The eight published cases show a striking preponderance in males and a predeliction for the mandible (Table I). (Note : In one report (Wunderer, 1953) only the location of the tumour is given.)

Table I.—*The Occurrence of CEOT from Published Data*

| Mean age at onset of symptoms | Sex* | | Location | |
|---|---|---|---|---|
| | Males | Females | Mandible | Maxilla |
| 33 years | 7 | 0 | 7 | 1 |

* Sex not stated in one case.

The first report of this neoplasm in a female and the second to be located in the maxilla is recorded in this paper.

### CASE REPORT

The patient, an African woman aged 35 years was first admitted to this hospital with a history of nasal stuffiness, nose bleeds and headaches for many years.

More recently she had become aware of a swelling over the right antrum. A mild degree of proptosis was noted at this time. Radiological examination showed the presence of dense opacities within the antrum together with erosion of the antral walls and extension of opacity beyond the lateral wall of the right antrum (Fig. 1). A radiological diagnosis of osteogenic new growth was made.

A biopsy was taken and reported as a simple odontome.

A year later the patient returned to hospital complaining of greater swelling and a more marked degree of proptosis. The maxillary antrum was thoroughly curetted. Histological examination showed the presence of an epithelial tumour with an acellular stroma, well marked intracellular change, the formation of small intraepithelial cysts containing eosinophilic material and abundant calcifications (Fig. 2). The tumour was considered to be identical with the calcifying epithelial odontogenic tumour (CEOT) described by Pindborg. A review of the first biopsy showed features essentially similar to those noted in the second.

On the strength of this diagnosis a thorough radiological examination of the cleared antrum was undertaken and an unerupted first molar was located in the floor of the antrum (Fig. 3). No attempt to remove this tooth was made, and no follow-up has been possible, as the patient has not returned and attempts at locating her have proved unsuccessful.

The calcifying epithelial odontogenic tumour appears to be relatively slow growing. In one case (Ivy, 1948), there was X-ray evidence indicating a very early lesion which was described as an enlarged follicle surrounding the embedded tooth. This was at an age when normal eruption of the tooth concerned (mandibular second premolar) had been delayed no more than about 2 years. Should this case be accepted as showing the earliest evidence of the tumour, then it took approximately another 10 years for clinical signs to develop. As has been indicated, the mean age for symptoms to appear is about 20 years after the age of expected eruption of the associated embedded tooth (normal age of eruption of the teeth concerned was 6–7 years in two cases, 17–21 years in one and 11–12 years in the rest).

As the tumour grows, it expands the surrounding bony structures and ultimately produces a noticeable swelling. Destruction of bone is a frequent X-ray finding. Invasion of soft tissues and medullary spaces by epithelial masses has been observed, so that in spite of its histologically degenerative appearance it is capable of causing considerable local destruction, as was observed in the present case.

In three of the reported cases the lesion was discovered on radiographic examination before obvious signs had appeared (Stoopack, 1957; Pindborg, 1958; Chaudhry, Holte and Vickers, 1962). In one, however, a diffuse enlargement developed 2 months after operation (Pindborg, 1958). In a fourth case swelling developed shortly after the removal of an embedded tooth at the age of 36 years, but it took another 6 years to cause discomfort and operation then was followed by recurrence 6 months later. The patient was operated on again and the tumour recurred once more after 11 years (Pindborg, 1958). Thus, because of its slow growth, recurrence of this tumour remains a possibility for many years. There is, in addition, some evidence that signs and symptoms are produced more rapidly after incomplete removal than with the original growth. Nor does it appear that extraction of the associated embedded tooth necessarily precludes the subsequent development of the tumour.

### MATERIALS AND METHODS

The specimen (formalin fixed) was divided into three samples: decalcified, undecalcified but containing gritty material, and tissue not requiring decalcification. Each sample was embedded in paraffin-wax and 10 serial sections from each block, cut at 4 micron intervals, were carefully examined. Some sections were studied by polarised light. Frozen sections were cut from the remainder. Selected sections were stained by a number of histochemical techniques and by less specific routine stains. The methods used and the results obtained are set out in Table II. Not recorded in the table is a von Kossa on undecalcified material

TABLE II.—*Staining Characteristics of the CEOT*

| Method | HS | Epithelial cell cytoplasm | Stroma* |
|---|---|---|---|
| H and E | red + | red + to +++ | red + |
| Van Gieson | yellow + | yellow ++ | red + |
| Masson's trichrome | green ++ | red | green ++ |
| Picro-Mallory | 0 to blue + | blue to red | blue +++ |
| Orcein | 0 | 0 | brown ± |
| Oil red 0 | 0 | 0 | 0 |
| Sudan IV | 0 | 0 | 0 |
| Silver method for glycogen & mucin (Gomori) | 0 | black | 0 |
| PAS | 0 to red + | red +++ | red + |
| PAS after diastase (McManus) | 0 to red + | 0 | red + |
| Chromic acid-Schiff (Bauer) | 0 | red + | 0 |
| Alcian blue | blue + | 0 | blue ++ |
| Mucicarmine (Southgate) | 0 | 0 | 0 |
| Toluidine blue | blue + | blue ++ | beta metachromasia |
| Amyloid | | | |
| (1) Methyl violet | violet + | violet +++ | violet + |
| (2) Congo red | orange + | orange + | orange + |
| Mercury-Bromphenol blue method for proteins (Bonhag, 1955) | blue | blue +++ | blue + to ++ |
| Millon reaction (Baker, 1956) | yellow to pink | pink | yellow to pink |
| DDD reaction for combined SS and SH groups (Barrnett & Seligman, 1952) | red + | red +++ | red + |
| Phosphotungstic acid-Haematoxylin (Mallory) | yellow to brown | dark blue fibrils | red to brown |
| Phloxine-Tartrazine (Lendrum) | red + | red ++ | yellow + |
| Methyl blue-Eosin (Mann) | violet + | violet +++ | blue ++ |
| Methyl green-Pyronin (Pappenheim) | 0 | red ++ | 0 |

Colour intensity

0 negligible
\+ weak
++ moderate
+++ strong

* Stroma in this instance refers only to tissue with a recognisable fibrous element.

which gave a strongly positive result and Gomori's alkaline phosphatase procedure which was attempted on a frozen section of stale tissue, with little success. The results obtained with Gordon and Sweet's silver reticulin method are described in the text.

Furthermore, special staining procedures were carried out on selected sections from 6 ameloblastomas and 2 craniopharyngiomas with calcifications to compare their epithelial and stromal changes with those of the CEOT.

### OBSERVATIONS

The epithelial component of the tumour consisted of sheets of polyhedral cells which tended to be closely packed in the advancing or infiltrating tumour edge (particularly apparent within the medullary spaces of osseous fragments (Fig. 4), and in the sub-epithelial connective tissue of the antrum) while in other parts there was separation of cells revealing prominent intercellular bridges (Fig. 5). In some fragments the tumour cells were associated with a narrow epithelial band consisting of three or four layers of flattened cells, which in some parts appeared separate from the tumour and in others more intimately associated with it. The impression obtained was that tumour cells were arising from this band in some areas (Fig. 6 and 7).

The nuclei of the tumour cells showed great variation in size and shape and while some were vesicular, the majority appeared hyperchromatic and pyknotic. Binucleate cells were frequent (Fig. 5) and some giant forms contained 3 or 4 nuclei. In some of the large, hyperchromatic nuclei a central unstained vacuole was seen. Mitotic figures were not observed.

The cytoplasm of the more compact cells contained glycogen and showed moderate pyroninophilia. Fibrils stained with phosphotungstic acid-haematoxylin were clearly demonstrated, particularly in cells which had become compressed

---

### EXPLANATION OF PLATES

FIG. 1.—Radiograph showing dense opacities filling the right antrum and destruction of the antral walls.

FIG. 2.—An area of the tumour showing multiple small intraepithelial cysts, focal calcifications and large conglomerates. H. & E. ×90.

FIG. 3.—Lateral view of cleared antrum revealing the embedded molar.

FIG. 4.—Compact masses of epithelial cells invading the medullary spaces of bone. H. & E. ×90.

FIG. 5.—Epithelium showing prominent intercellular bridges. Note pyknotic nuclei and binucleate cells. Arrow indicates cell with intensified cytoplasmic eosinophilia H. & E. ×340.

FIG. 6.—A general view of the tumour showing epithelial islands in a relatively acellular stroma. Arrow indicates probable reduced enamel epithelium. H. & E. ×20.

FIG. 7.—Higher power view showing relationship of tumour to the narrow epithelial band. H. & E. ×134.

FIG. 8.—Spiral shaped fibrils within the cytoplasm of tumour epithelial cells. P.T.A.H. ×840.

FIG. 9.—Epithelial cells showing delicate intracytoplasmic birefringence when viewed by polarising light. H. & E. ×340.

FIG. 10.—High-powered detail of epithelium to show intracellular origin of HS. Note multinucleate cells. H. & E. ×525.

FIG. 11.—Intraepithelial accumulations of HS producing a honeycombed multi-cystic appearance. Note coarse reticulin fibres around small blood vessels and larger accumulations of HS. Gordon and Sweet's silver reticulin ×145.

FIG. 12.—" Melting down " of epithelium to produce a pool of HS containing nuclear debris. H. & E. ×145.

FIG. 13.—Loose and fragmented stromal reticulin fibres showing tendency to accommodate droplets of HS. Gordon and Sweet's silver reticulin ×145.

FIG. 14.—A. indicates the subepithelial connective tissue of the antrum and B. the infiltrating tumour edge. Compare the loose reticulin pattern of tissue infiltrated by tumour cords with the dense reticulin pattern of the former. Gordon and Sweet's silver reticulin ×20.

1

2

3

4

Gon.

Gon.

11

12

13

14

Gon.

and assumed a more elongated shape They tended to have a spiral form surrounded the nucleus in a plane parallel to the long axis of the cell and extended to the cell borders. With polarised light a delicate intraepithelial birefringence was observed (Fig. 8 and 9). From these findings it would appear that the cell type is identical with that of the stratum spinosum of the epidermis and comparable to the cells making up the stratum intermedium of the enamel organ.

Some cells showed an intensification of cytoplasmic eosinophilia, which in some instances seemed to precede the development of an intracytoplasmic homogeneous substance (HS) (Fig. 10). This HS expanded the cell borders and produced large, globular cells with crescentic nuclei. The presence of small discrete globules in some of the larger cells tended to give them a foamy appearance. Ultimately the cell walls ruptured with spillage of this material which then spread intercellularly, separating cells and uniting with other globules to form small pseudocysts in the epithelial islands (Fig. 11). As this process continued the epithelium " melted away " leaving a pool of HS in which could be seen a sprinkling of nuclei and nuclear debris (Fig. 12). The larger accumulations of HS became vascularised by an ingrowth of delicate capillaries.

These pools formed an acellular background stroma to the tumour, which has been referred to as " dense hyaline tissue " (Ivy, 1948). It stains weakly with connective tissue stains and yellow with Van Gieson's stain. Collagenous tissue, as indicated by red staining with the latter technique and bright blue with Mallory's aniline blue stain, was confined to irregular islands around a few of the larger vessels and some of the calcified conglomerates.

Although no reticulin could be demonstrated in the small globules some of the larger accumulations were surrounded by coarse and fragmented reticulin fibres (Fig. 11) which combined to give the background stroma a loose reticulin pattern with a tendency for the fibres to be arranged in a circular or ovoid manner as if to accommodate globules of HS which could in fact be demonstrated in a few foci (Fig. 13). This loosening and fragmentation was readily apparent where the tumour was found infiltrating nasal subepithelial connective tissue, and compared to the relatively dense reticulin pattern of the latter. Weakening of collagen staining and loosening of reticulin was often observed for a short distance in advance of the infiltrating epithelial cells (Fig. 14). The background of HS was best differentiated from the surviving true connective tissue stroma by careful differentiation with phloxine tartrazine or by staining with a mixture of the two anionic dyes eosin and methyl blue.

Mineralisation in some instances commenced while the homogeneous substance was still located within the cell. It was appositional in nature and exhibited Liesegang's rings. Large conglomerates containing calcium phosphate were eventually formed (Fig. 2). The largest ones were surrounded by collagenous connective tissue and were situated in what appeared to be the " burnt out " parts of the lesion. This agrees with Pindborg's (1958) comment that the older the tumour the more pronounced the calcification. Structures resembling dentine, described by Chaudhry, Holte and Vickers (1962), were not found.

One of the interesting aspects of this tumour is the development of an intracellular homogeneous substance (HS) which ultimately produces dissolution of the cell. It has been termed a " degenerative " change and is regularly mineralised. Degenerative changes with dystrophic calcification occur fairly frequently in a wide variety of lesions. However, an ectodermal tumour which in all the cases described has produced an amorphous material that is avidly mineralised raises the question of whether it might not represent an attempt by the cells to carry out a function, the result of which, under developmental conditions, is a matrix destined for calcification by intention.

### *A Histochemical Comparison of the Tumour with Developing Enamel, the Ameloblastoma and the Craniopharyngioma*

In view of what has been stated, an attempt to discover the nature of HS was made by comparing it with the normal products of the dental organ epithelium. Should the tumour be attempting to recover the function of the original enamel organ, then apart from inductive changes in the pulp, it would be responsible for the elaboration of: (1) The gelatinous intercellular substance of the stellate reticulum; (2) enamel matrix; (3) primary enamel cuticle. However, should it represent an attempt to carry on the function of the reduced enamel organ then it is responsible for (1) the secondary enamel cuticle; (2) a possible desmolytic enzyme.

*Stellate reticulum.*—The ground substance of the stellate reticulum has been described as a mucoid fluid rich in albumin (Orban, 1957) and acid mucopolysaccharides (Wislocki and Sognnaes, 1950; Bevelander and Johnson, 1955; Sasso and Castro, 1957). Staining of HS for acid mucopolysaccharides yielded negative results with the methods employed. Recently some doubt has arisen regarding the presence of acid mucopolysaccharides in the stellate reticulum (Shear, personal communication), but the author has obtained positive results for acid mucopolysaccharides in the stellate reticulum-like areas of some ameloblastomas, particularly those undergoing cystic change.

*Enamel Matrix.*—Although in no part of the tumour did the epithelial cells have any resemblance to those of the ameloblastic layer, the morphologically identical cells of the stratum intermedium are thought to have an important role in the development of enamel and take an active part in the calcium metabolism of the inner dental epithelium (Orban, 1957). Furthermore, it has been stated by many that enamel formation does not occur in ameloblastomas. Boyle and Kalnins (1960), however, examined 17 ameloblastomas and concluded that amelogenesis could be observed in 6, mainly in the form of transitional and semi-mineralised droplets. The staining reactions of pre-enamel and young enamel as given by Shear (1962) are compared with HS in Table III, and from these it

TABLE III.—*Some of the Staining Reactions of HS Compared with Enamel Matrix*

| Method | HS | Pre-enamel | Young enamel |
|---|---|---|---|
| H and E | E | E | E |
| Reticulum | — | — | — |
| Mucicarmine | — | + | — |
| PAS | — | — | — |
| Alcian blue | + | + | — |
| Toluidine blue | — | — | — |
| Van Gieson | yellow | light brown | dark brown |
| Mallory | blue + | blue + | orange |

would appear that there are only minor differences. The similarity, however, is mainly due to the negative reactions obtained with the methods employed. The droplets observed in ameloblastomas by Boyle and Kalnins (1960) stained red with Mallory's aniline blue stain in the young-enamel stage and gave a strong blue colour in the transitional stage.

There is, in addition, chemical and histochemical evidence that a form of keratin is produced in the process of elaborating enamel matrix (Stack, 1955). Positive sulphydril (SH) reactions which are replaced in part by a reaction for disulphide groups (SS) have been observed in ameloblasts and pre-enamel (Sognnaes, 1955). HS yielded negative results when stained by the Barrnett and Seligman (1952) DDD method for combined SS and SH groups, whereas the cornifying areas in an ameloblastoma with squamous metaplasia and an epidermal tumour which were stained as controls gave positive reactions. No relationship between HS and enamel matrix could be established from its reactions with the histochemical method for SH and SS groups or the less specific stains such as Masson's trichrome and Mallory's connective tissue stain. The possibility that it may be an altered form of pre-enamel cannot be entirely ruled out.

*The primary enamel cuticle* is formed as the last product of the ameloblasts and is intimately associated with enamel matrix of the fully formed tooth (Toller, 1948 ; Ussing, 1955). It is considered that the evidence against enamel matrix will in the main apply to the primary enamel cuticle.

*The secondary enamel cuticle* (attachment cuticle) is formed as the tooth erupts (Toller, 1948). It is a non-cellular keratinised layer elaborated by the epithelial attachment of the tooth (formerly reduced enamel epithelium) (Orban, 1957). Although the CEOT is associated with unerupted teeth the possibility exists that with failure of eruption a tumour of reduced enamel epithelium might nevertheless attempt to form this material. For this reason some of the staining results were compared with procedures carried out on the secondary enamel cuticle by Wertheimer and Fullmer (1960, 1962) and Fullmer and Wertheimer (1960) (Table IV).

TABLE IV.—*Some of the Staining Reactions of HS Compared with the Secondary Enamel Cuticle*

| Method | HS | Cuticle |
|---|---|---|
| Mallory . . . . | 0 to blue + | . red |
| Orcein . . . . | 0 | . brown |
| PAS . . . . . | 0 to red + | . 0 |
| Chromic acid-Schiff (Bauer) . | 0 | . red |
| Silver method for glycogen and mucin (Gomori) | 0 | . black |
| DDD reaction for SS and SH groups | red + | . blue |

From this comparison no apparent histochemical relationship to the fully formed secondary enamel cuticle can be deduced.

*A desmolytic enzyme.*—As the crown of the erupting tooth moves towards the surface, the connective tissue between the reduced enamel epithelium and the oral epithelium disappears and it is thought that the degeneration of the fibres is due to an enzymatic action by the proliferating epithelial cells (Orban, 1957). Ussing (1955) observed that in the dental sac the connective tissue varies from " closely packed collagen fibres to a loose myxomatous type in the path of eruption ". She thought that the depolymerisation of the connective tissue ground substance may be due to a mucolytic enzyme secreted by the epithelial cells.

From observations in this study it was concluded that there was considerable stromal change, particularly loss of collagen ground substance and destructive alterations in the reticulin network. Furthermore, this degeneration was intimately related to the epithelial tissue and could be observed where the epithelium had invaded para-nasal connective tissue.

It has often been noted, however, that stromal changes occur not infrequently in ameloblastomas (Lucas, 1957*a*). Cysts may form (Kramer, 1963) and all stages between loosening of connective tissue to mucinous degeneration and the formation of completely clear spaces may be observed (Lucas and Thackray, 1951 ; Cooke and Harrison, 1955). To compare them with the stromal changes in the calcifying epithelial odontogenic tumour, 6 ameloblastomas exhibiting well marked stromal changes were studied by staining with Van Gieson's, Masson's trichrome, toluidine blue, PAS after diastase, alcian blue and the silver reticulin technique of Gordon and Sweet. The results obtained were essentially similar in all 6 cases, namely, a loss of red collagen staining with van Gieson's stain and a decrease in intensity of green staining with Masson's trichrome in areas where morphological stromal alterations were apparent. These areas exhibited an increasing metachromasia with toluidine blue and usually stained more intensely with PAS after diastase and alcian blue. With cystic degeneration the contained fluid reacted strongly with alcian blue, often showed gamma metachromasia, but usually only stained weakly with PAS. The reticulin pattern showed no gross alterations apart from compression of fibres where cystic fluid accumulations had taken place.

Various changes in some ameloblastomas can result in areas resembling a cylindroma (Rewell, 1963) and a number of ameloblastomas with eosinophilic hyaline material around and within epithelial islands were studied. These tended to resemble salivary gland cylindromas and some areas had a superficial likeness to the CEOT. However, they were distinguished from the stromal changes in the CEOT by the alcian blue, PAS after diastase and toluidine blue reactions. The results obtained were comparable to those given by the " hyaline material " of adenoid cystic salivary gland carcinomas (Azzopardi and Smith, 1959).

It would appear from this study that the stromal changes observed in the CEOT are fairly unique and, unlike those of ameloblastomas, are not associated with an increase in mucopolysaccharides. The stromal changes would be best explained by a desmolytic enzyme producing destruction of collagen and reticulin, which is replaced in part by accumulations of HS.

*Calcifications* are not commonly found in odontogenic epithelial tumours and cysts. The only other odontogenic epithelial tumour in which calcification almost invariably occurs is the adeno-ameloblastoma. Bernier and Tiecke (1956) reported its presence in all their 9 cases (it is also of interest that 6 of these cases were associated with impacted teeth or follicular cysts). Oehlers (1956), reporting on an adeno-ameloblastoma which arose in the wall of a dentigerous cyst, concluded after a careful study that the calcified bodies were associated with epithelial cells and not with connective tissue. Willis was of the opinion that the calcified masses resembled salivary stones.

Villa and Bunag (1956) described a tumour which they called a soft mixed odontome and observed that in some of the epithelial masses the cells next to the ameloblast-like layer arranged themselves like an exaggerated stratum intermedium and underwent calcification. Villa (1951) also reported a case of calcification of the reduced enamel epithelium and of the epithelial remnants in the follicle of an embedded molar. He concluded that these calcified bodies were definitely the result of calcific degeneration of the epithelial cells.

Lucas (1957*b*) in an article on an adeno-ameloblastoma remarked that the scattered foci of calcification represent local depositions of calcium in dead or degenerating epithelial cells such as sometimes occur in the epithelial rests in the periodontal membrane.

Ussing (1955) in a study on the reduced enamel epithelium stated that in several cases strands of epithelial cells could be seen penetrating the connective tissue from the tooth side of the dental sac. The epithelial cells had deeply stained nuclei and degeneration of these cells resulted in the formation of calcified bodies.

In a series of 80 odontogenic tumours and cysts from this Institute only one epithelial lesion with calcifications was found, and that within a dentigerous cyst. In view of the fact that calcification is described as characteristic in the histologically similar craniopharyngioma (Kernohan and Sayre, 1956 ; Gorlin, Chaudhry and Pindborg, 1961), 12 tumours were studied. Four were found to contain calcification. In each instance there was an attempt at cornification (strong positive result with the DDD reaction for combined SH and SS groups) which was preceded by the formation of large, somewhat flattened " ghost cells ". Calcification, when it occurred, was within these keratin-like masses. The histological features were identical with those of the calcifying odontogenic cyst described by Gorlin, Pindborg, Clausen and Vickers (1962), who had also called these tumours " oral Malherbe ", and also bore a striking resemblance to the only calcifying epithelial lesion found in this series.

It was thus concluded that calcification, though not uncommon in reduced enamel epithelium, is a rare occurrence in odontogenic epithelial tumours and when it does occur, it appears to be invariably associated with degenerations or transformations of the epithelial cells which are often of a cornifying nature, and not a consequence of stromal change.

It is of interest to record that the stratum intermedium, which is thought by some to be the dominant element in the reduced enamel epithelium (Johnson and Bevelander, 1957), is rich in alkaline phosphatase (Sasso and Castro, 1957).

### HISTOGENESIS

Any attempt to determine the histogenesis of the CEOT must be related to its constant association with an unerupted permanent tooth. The association of ameloblastomas with unerupted teeth never shows this constancy. Robinson (1937) in a review of 379 cases of ameloblastoma records only 14 tumours associated with unerupted teeth.

As no remnants of the enamel organ remain within the tissue of the fully erupted tooth (Rewell, 1963) the implication is that the reduced enamel epithelium is probably intimately involved in the origin of the tumour. This view has been put forward by Pindborg (1958) and supported by Chaudhry, Holte and Vickers (1962). The results of the serial sections in the study undertaken here suggest an origin from a structure histologically compatible with the reduced enamel epithelium. In the case described by Ivy (1948) there is X-ray evidence from 10 years before the onset of clinical signs (at age of 14 years) of a somewhat enlarged follicle surrounding the crown of the second premolar. Nothing further was done at that stage because it was felt that the position of the tooth was good and normal eruption would take place in due course. Ivy concluded that instead, abnormal proliferation of the epithelial cells occurred and subsequently resulted in a new growth.

When the enamel has completely matured the ameloblasts degenerate and can no longer be differentiated from the cells of the stratum intermedium and the outer dental epithelium, and form a stratified epithelial covering for the enamel. Johnson and Bevelander (1957) studied the canines of foetal pigs and showed that prior to the engagement of the erupting tooth with the oral mucosa, the stratum intermedium proliferates into a multicellular layer overlaid by degenerating ameloblasts and that it is in " its ascendence while the ameloblastic layer is degenerating ". They concluded that the epithelial attachment of the tooth is a product of the stratum intermedium. The histological resemblances of the epithelial cells of this tumour to those of the stratum intermedium of the enamel organ were commented on above.

It is therefore postulated that this tumour is the rare consequence of continued proliferation by the cells of the reduced enamel epithelium and in particular those of the original stratum intermedium, in an attempt to carry out their normal function of fusion with the oral epithelium when the tooth for some reason fails to erupt. It should be mentioned that the X-ray appearances of the lesion of 4 of the cases (Thoma and Goldman, 1946 ; Ivy, 1948 ; Stoopack, 1957 ; and Chaudhry, Holte and Vickers, 1962), were interpreted as a dentigerous cyst, while in one of; Pindborg's cases it is reported that a dental cyst was excised at one stage. The transformation of a cyst into an ameloblastoma is not uncommon (Thoma and Goldman, 1960). Cahn (1933) has called the dentigerous cyst a potential adamantinoma and Bernier (1960) reports a 33 per cent association of ameloblastomas with follicular cysts. There is a likelihood, therefore, that in some cases the tumour may develop from a pre-existing dentigerous cyst (Fig. 15).

FIG. 15.—Illustrates the possible origin of the tumour from a dentigerous cyst.

In spite of the infiltrative nature of the tumour the cell nuclei show considerable degenerative changes. In addition, there is an unusual homogeneous intracytoplasmic change which results in destruction of the cell. The results of histochemical and non-specific staining procedures show that it is an amorphous, weakly acidophilic substance of low protein content, which is permeable to ion-aggregates but less so than collagen and has a strong affinity for mineral salts, particularly calcium phosphate. It has not been possible to establish any relationship to the products of either the enamel organ or the reduced enamel epithelium and unless new evidence is supplied, possibly by enzyme studies on fresh material, it must be considered an unusual form of epithelial degeneration. The connective tissue changes associated with this tumour, however, suggest the possible production of a desmolytic enzyme which may to some extent explain its behaviour.

The view held by some (Bernier, 1960 ; Thoma and Goldman, 1960) that the tumour is a calcifying variant of an ameloblastoma is not borne out by the findings of this investigation. The views of the present author are in agreement with its inclusion as a distinctive epithelial tumour in Pindborg and Clausen's (1958) classification of odontogenic tumours.

The cell type is morphologically identical with the cells of the stratum spinosum of the epidermis, the stratum intermedium of the enamel organ and the reduced enamel epithelium. The tumour can be considered an odontogenic acanthoma.

Lesions imitating the ameloblastoma have been seen in sebaceous cysts, the tibia, parapituitary residues and salivary glands (Willis, 1960). It would be of interest to know if this tumour has been mimicked in other sites.

### SUMMARY

(1) The first case of a calcifying epithelial odontogenic tumour in a female and the second to be located in the maxilla, is recorded.

(2) A survey covering a 10-year period and 80 odontogenic lesions confirmed the rarity of this tumour.

(3) A wide variety of special stains including histochemical procedures were used in an attempt to elucidate the nature of the lesion. Certain aspects of ameloblastomas and calcifying craniopharyngiomas were studied and compared to it.

(4) The results indicate that the tumour arises either directly from the reduced enamel organ or possibly from a dentigerous cyst. Attempts at relating certain aspects of the tumour to products of the enamel organ and the reduced enamel epithelium were unsuccessful. The production of a desmolytic enzyme is postulated.

My thanks are due to the Superintendent of the Far East Rand Hospital for permission to publish this case, the Director of the South African Institute for Medical Research for facilities granted, Dr. N. S. F. Proctor, Dr. D. Goldstein and Dr. M. Shear for suggestions and advice and Miss M. Peterson and Miss D. Scarrott for their technical assistance. The photographic work was done by Mr. M. Ulrich.

### REFERENCES

AZZOPARDI, J. G. AND SMITH, O. D.—(1959) *J. Path. Bact.*, **77**, 131.

BAKER, J. R.—(1956) *Quart. J. micr. Sci.*, **97**, 161.

BARRNETT, R. J. AND SELIGMAN, A. M.—(1952) *J. nat. Cancer Inst.*, **13**, 215.

BERNIER, J. L.—(1960) ' Tumors of the odontogenic apparatus and jaws '. Washington D.C. (Armed Forces Institute of Pathology).

*Idem* AND TIECKE, R. W.—(1956) *Oral Surg.*, **9**, 1304.

BEVELANDER, G. AND JOHNSON, P. L.—(1955) *J. dent. Res.*, **34**, 123.

BONHAG, P. F.—(1955) *J. Morph.*, **96**, 381.

BOYLE, P. E. AND KALNINS, V.—(1960) *Arch. oral Biol.*, **2**, 285.

CAHN, L. R.—(1933) *Dent. Cosmos*, **75**, 889.

CHAUDHRY, A. P., HOLTE, N. O. AND VICKERS, R. A.—(1962) *Oral. Surg.*, **15**, 843.
COOKE, B. E. D. AND HARRISON, D. F. N.—(1955) *Brit. dent. J.*, **98**, 159.
FULLMER, H. M. AND WERTHEIMER, F. W.—(1960) *Stain Tech.*, **35**, 156.
GORLIN, R. J., CHAUDHRY, A. P. AND PINDBORG, J. J.—(1961) *Cancer, Philad.*, **14**, 73.
*Idem*, PINDBORG, J. J., CLAUSEN, F. P. AND VICKERS, R. A.—(1962) *Oral Surg.*, **15**, 1235.
IVY, R. H.—(1948) *Ibid.*, **1**, 1074.
JOHNSON, P. L. AND BEVELANDER, G.—(1957) *Ibid.*, **10**, 437.
KERNOHAN, J. W. AND SAYRE, G. P.—(1956) ' Tumors of the pituitary gland and infundibulum '. Washington D.C. (Armed Forces Institute of Pathology).
KRAMER, I. R. H.—(1963) *Brit. J. oral Surg.*, **1**, 13.
LUCAS, R. B.—(1957*a*) *Oral Surg.*, **10**, 863.—(1957*b*) *Ibid.*, **10**, 652.
*Idem* AND THACKRAY, A. C.—(1951) *Brit. J. Cancer*, **5**, 289.
OEHLERS, F. A. C.—(1956) *Oral Surg.*, **9**, 411.
ORBAN, B. J.—(1957) ' Oral histology and embryology '. London (Henry Kimpton).
PINDBORG, J. J.—(1958) *Cancer, Philad.*, **11**, 838.
*Idem* AND CLAUSEN, F. P.—(1958) *Acta odont. scand.*, **16**, 293.
REWELL, R. E.—(1963) ' Pathology of the upper respiratory tract '. Edinburgh (Livingstone)
ROBINSON, H. B. G.—(1937) *Arch. Path.*, **23**, 831.
SASSO, W. S. AND CASTRO, N. M.—(1957) *Oral Surg.*, **10**, 1325.
SHEAR, M.—(1962) *Brit. dent. J.*, **112**, 494.
SOGNNAES, R. F.—(1955) *Ann. N.Y. Acad. Sci.*, **60**, 545.
STACK, M. V.—(1955) *Ibid.*, **60**, 585.
STOOPACK, J. C.—(1957) *Oral Surg.*, **10**, 807.
THOMA, K. H. AND GOLDMAN, H. M.—(1946) *Amer. J. Path.*, **22**, 433.—(1960) ' Oral pathology, 5th edition, St. Louis (Mosby).
TOLLER, J. R.—(1948) *Brit. dent. J.*, **84**, 255.
USSING, J. M.—(1955) *Acta odont. scand.*, **13**, 123.
VILLA, V. G.—(1951) *Oral Surg.*, **4**, 877.
*Idem* AND BUNAG, C. A.—(1956) *Ibid.*, **9**, 1218.
WERTHEIMER, F. W. AND FULLMER, H. M.—(1960) *J. Histochem. Cytochem.*, **8**, 442.—(1962) *J. Periodont.*, **33**, 29.
WILLIS, R. A.—(1960) ' Pathology of tumours ', 3rd edition, London (Butterworth).
WISLOCKI, G. B. AND SOGNNAES, R. F.—(1950) *Amer. J. Anat.*, **87**, 239.
WUNDERER, S.—(1953) *Ost. Z. Stomat.*, **50**, 567.